# Being in the right place at the right time

**DOI:** 10.1093/sleepadvances/zpaf033

**Published:** 2025-05-21

**Authors:** Sonia Ancoli-Israel

**Affiliations:** Department of Psychiatry, University of California, San Diego, CA, United States

**Keywords:** actigraphy, light therapy, periodic limb movements, OSA, sleep apnea, aging, neurodegenerative disorders, Alzheimer’s disease, cancer, circadian rhythms

## Abstract

This paper is a review of my life as a sleep researcher and clinician. I chanced into sleep by being at the right place at the right time. Over the last 45 years, I became an expert on sleep and circadian rhythms in aging, in Alzheimer’s disease and Parkinson’s disease and in cancer. We were one of the first to show how common sleep apnea and periodic limb movements in sleep are in the elderly. We “moved” into the nursing home and showed how disrupted sleep is in these patients. We used light therapy to improve sleep in the nursing home. We studied the effect of CPAP on sleep and cognition in both Alzheimer’s disease and Parkinson’s disease. And we were some of the first to study sleep and circadian rhythms in women with breast cancer, starting the evaluations before they started their chemotherapy. These studies were both observational and treatment studies. I am very proud of the work we did. For me, everything revolves around sleep. And that is the beginning of my story.

I was born on Christmas Day, outside of Tel Aviv, Israel, to survivors of the Holocaust. There was little family left after the Holocaust, so my birth was a testament to survival. At the age of 3, my father’s career took us from Jerusalem to New York City. We ended up staying in New York and that’s where I grew up.

When it was time for High School, I applied to the High School of Music and Art (now called Fiorello H. LaGuardia High School of Music & Art and Performing Arts) because my older sister, Annie, went there. But I also applied to the Bronx High School of Science, another specialized public high school in New York. When my letter came from Bronx Science, I cried. My mother tried to comfort me, saying it was OK that I didn’t get in. But I did get in. I was crying because I realized that I was not meant to be a musician. Science was my love and I was meant to be a scientist. And thus began my career.

## Research in Psychophysiology and Biofeedback

When it was time for college, I did follow in my sister’s footsteps and ended up at the State University of New York, Stony Brook (now called Stony Brook University). I started out pre-med but organic chemistry did me in. So, I switched to psychology. In my sophomore year, my Resident Assistant was Patricia Cowings, who as an upper class(wo)man, was working in the biofeedback laboratory of Dr. Lester Fehmi. Pat and Les invited me to work in the lab with them and that is really where my career as a researcher began. As an aside, Pat always wanted to be an astronaut, and she ended up being the first American woman to be trained as a scientist-astronaut by NASA. While an alternate for the 1979 space flight, she never did make it to space, but she did study the effects of gravity on human physiology and performance and made her mark there.

Pat and Les introduced me to alpha biofeedback training. We hooked up our subjects to a Beckman polygraph, which Pat had affectionately named Aloysius, a name meaning a “famous warrior. I have no idea why she chose that name. I spent many hours standing in front of Aloysius recording EEG data. I learned how to place electrodes. I learned how to read an EEG. I learned how to use a polygraph. I learned to wear a white coat and step back when the pens went crazy spewing ink everywhere. But mostly I learned how to do good research in psychophysiology. My first published abstract came from this work, titled, “Effectiveness of tactile and auditory feedback on the self-regulation of frontal lobe EEG activity” [1].

I had decided to become a clinical psychologist and in my senior year of college applied to clinical psychology PhD programs. I had excellent grades and test scores. But in those days, it was difficult for women to get into PhD clinical psychology programs, and I was rejected from all of them. (Times have really changed as most PhD clinical psychology programs are primarily women now). So, I applied to master’s programs. The only schools that had not reached their deadlines were the California State University schools. My sister was living in Los Angeles, so I followed her again, made the move and entered the psychology master’s program at California State University, Long Beach. Since I had experience doing research in psychophysiology, I joined the laboratory of my next mentor, Dr. Kenneth Green, where I continued to do research in biofeedback. My first publication, based on my master’s thesis, was “Authoritarianism, introspection and alpha wave biofeedback training” [2] I screened 50 college-age volunteers, and gave them paper and pencil tests to rate the traits of introspection and authoritarianism. Seven subjects from each extreme of the resulting scales then underwent six alpha wave biofeedback training sessions, conducted in a darkened room with eyes closed. The results showed that those scoring high in introspection and low in authoritarianism could produce a larger difference between their alpha-on and alpha-off periods (thus indicating control), than those scoring low in introspection and high in authoritarianism.

I always knew I wanted to continue on to get a PhD and this time in experimental psychology, continuing my work in biofeedback and psychophysiology. While in my last year of my master’s program, I saw an announcement that Dr. Joe Kamiya would be speaking at UCLA, on Yom Kippur, the most sacred of all the Jewish holidays. I debated whether to go hear him or not and decided God would forgive me. Afterall, Joe was the father of biofeedback. He started the whole field, and I just could not miss the opportunity to meet him.

So I drove up from Long Beach to UCLA. Before he started speaking, I went up to introduce myself and told him I had worked with Les Fehmi (biofeedback was a small field and the researchers all knew each other). We chatted for a few moments about Les and about my work. Then Joe began his talk. The first thing he said was, “By the way, can anyone give me ride back to the airport?” My hand shot up. I was going right past the airport anyway, but I would have volunteered even if it had been miles out of my way.

While in the car, Joe asked me about my future plans. I mentioned that I wanted to get a PhD, and he invited me to apply to study and work with him in the PhD psychology program at the University of California, San Franciso where he was a professor and ran a biofeedback lab. And as they say, the rest is history. Being in the right place at the right time.

I moved to San Francisco and studied with Joe for five years as I completed my PhD. My dissertation was with Joe and Dr. Paul Ekman on psychophysiological response patterns of emotion [3, 4]. As part of the PhD program, we had to choose four areas of specialty. One of my areas was sleep and I was fortunate enough to learn about sleep from Dr. Irwin (Bob) Feinberg, a pioneer in the field of sleep. I already could read an EEG, but I learned how to score sleep stages and read polysomnograms. I remember that we would sit together, and he would talk and teach me. One day I started dozing off, and he kicked my foot to wake me back up. Ah, the sleep-deprived life of a graduate student.

As an aside, years later I was able to combine my interest in biofeedback and sleep by studying the effects of thermal biofeedback on periodic limb movements in sleep [5].

When I graduated with my PhD, I already had 9 publications. Not a bad start as I went on to my next chapter, a move to San Diego. But first some background as to why San Diego.

While I was finishing my master’s degree at Cal State Long Beach, my father was diagnosed with a glioblastoma. In April of that year, I flew back to New York to see my dad, and the medical student on his case stopped by to see him. That was Andrew Israel. We had dinner together, in the hospital, and then I flew back to California. But when I graduated, I drove back to New York to spend my dad’s last days with him. Andy and I started dating, although to be honest, our dates consisted of sitting at my father’s bedside. My dad died that July at age 52. And Andy was my biggest support.

**Figure 1.**
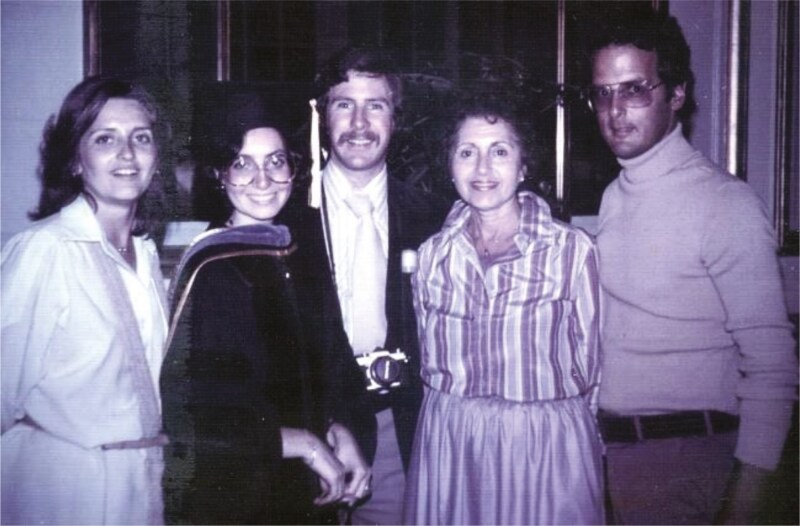
My PhD graduation in 1979 with my sister Annie, me, my soon-to-be husband Andy, my mom Esther and my brother-in-law Gary.

But I was on my way to San Francisco, and he had two more years of medical school in New York, so we began what turned into a 3.5-year long-distance relationship. When he graduated, he moved to San Diego to be closer to his family (he was a California boy) and so, in 1978, when I finished my data collection for my dissertation, I moved down to San Diego as well. As I was finishing writing my dissertation, I took a part-time job back at the California State University in Long Beach, my alma mater, teaching psychophysiology to undergraduate students. I graduated with my PhD in 1979 (see  Figure 1) and Andy and I got married a few months later. Now it was time to find a full-time position in San Diego.

## Sleep in Aging

I first went to visit Dr. Laverne “Verne” Johnson at the Naval Health Research Center. Verne was one of the founders of sleep research and was among the small group of researchers who, in 1969, drew up the definitions of the stages of sleep (and some of those early researchers have their own Living Legends papers). But Verne had just hired a new postdoc, Dr. Cheryl Spinweber, who later became a major sleep figure in her own right (and has her own Living Legends paper).

My next stop was to see Dr. Daniel Kripke at the University of California, San Diego. I met with Dan. We talked for a while. He was impressed that I had studied with Bob Feinberg and that among my publications were two papers on computer rejection of EEG artifacts, published with Dr. Alan Gevins. Dan told me he had no money, but he had a desk and a phone. (Back then there was no money but there was space. Today there is no money and no space!). He invited me to sit at that desk and write a grant.

NIH had just put out an RFA (request for applications) on sleep and aging. This was in 1980, and little was known about the clinical aspects of sleep and aging. So I wrote a grant proposing to look at the prevalence of sleep apnea and periodic leg movements in sleep (PLMS) (somewhat newish disorders) in a community sample of older adults. The study was funded, and I was off and running. My career path was determined by Dan giving me a desk, having faith in me, and the funding of that first grant. And again, the rest is history. And being at the right place at the right time.

**Figure 2.**
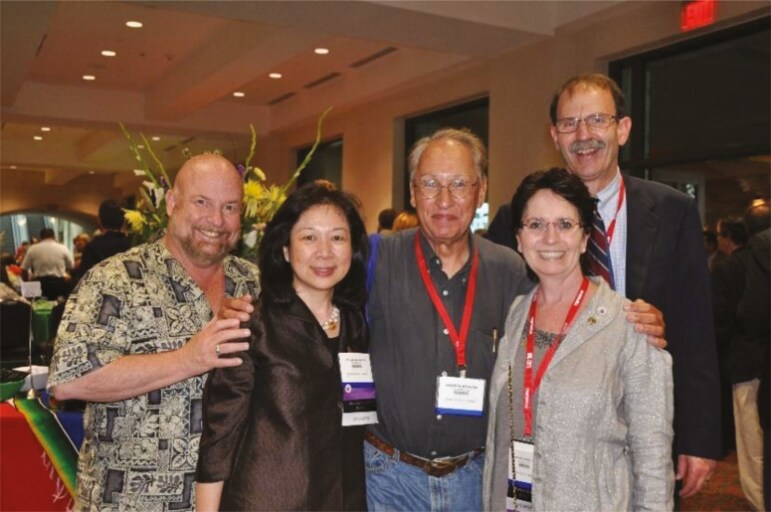
The “aging” cohort in 2010: Michael Vitiello, Phyllis Zee, Andy Monjan, Sonia, Don Bliwise.

I became one of just a handful of investigators studying older adults with that group including Don Bliwise, Pat Prinz, and Michael Vitiello—my first “aging” colleagues. Later Phyllis Zee joined the group. And over time others studied aging including Tim Monk, Eus van Someren, and others. While we are all in the same area of sleep research, and while our research was often quite parallel, we weren’t competitive; rather I have many fond memories of our many discussions about sleep and aging (see Figure 2).

My funded study was one of the first in many respects. It was the first large-scale study of sleep disorders in older adults. We screened over 600 adults over the age of 65 years and studied over 400 of them. We thought we were conducting an epidemiological study with that many subjects. But years later, when real card-carrying epidemiologists like Drs. Terry Young and Katie Stone entered the field of sleep and studied thousands of adults, we realized what true epidemiology is.

Our study was, however, one of the first, if not the first, to use home sleep recordings and actigraphy to study sleep. We used the modified Respitrace/Medilog portable system, an analog recorder that recorded data on a cassette tape that was then played back onto a polygraph. We measured thoracic and abdominal respiration, leg movements, and wrist activity to estimate wake and sleep [6, 7].

And our results? We found that sleep apnea and PLMS were both extremely common in older adults. At the time, hypopneas had not been defined yet, so our initial work was based just on the number of apneas per hour of sleep. The result was a prevalence rate of 24% for sleep apnea and 45% for PLMS. Later, when hypopneas were defined, we went back and re-scored all 427 records which showed that the prevalence of sleep apnea when using AHI was closer to 62% [8, 9] It was gratifying when those larger epidemiological studies of thousands of participants conducted by Drs. Terry Young, Katie Stone, and Susan Redline confirmed our results.

## Sleep in the Nursing Home

Around that same time, Dr. Elizabeth Barrett-Connor invited me to be part of a Teaching Nursing Home (TNH) Program project. The TNH program was originally funded by the Robert Wood Johnson Foundation. The mission was to improve the quality of clinical care in nursing homes and promote clinical research. The NIH National Institute on Aging (NIA) then began funding TNH research programs and Elizabeth wanted to include sleep as one of the projects. That’s where I came in. The UCSD TNH was funded, and I was PI of a project entitled “Effect of Hypnotics on Sleep Apnea in Nursing Home Patients.” And thus began my research in nursing home patients.

**Figure 3.**
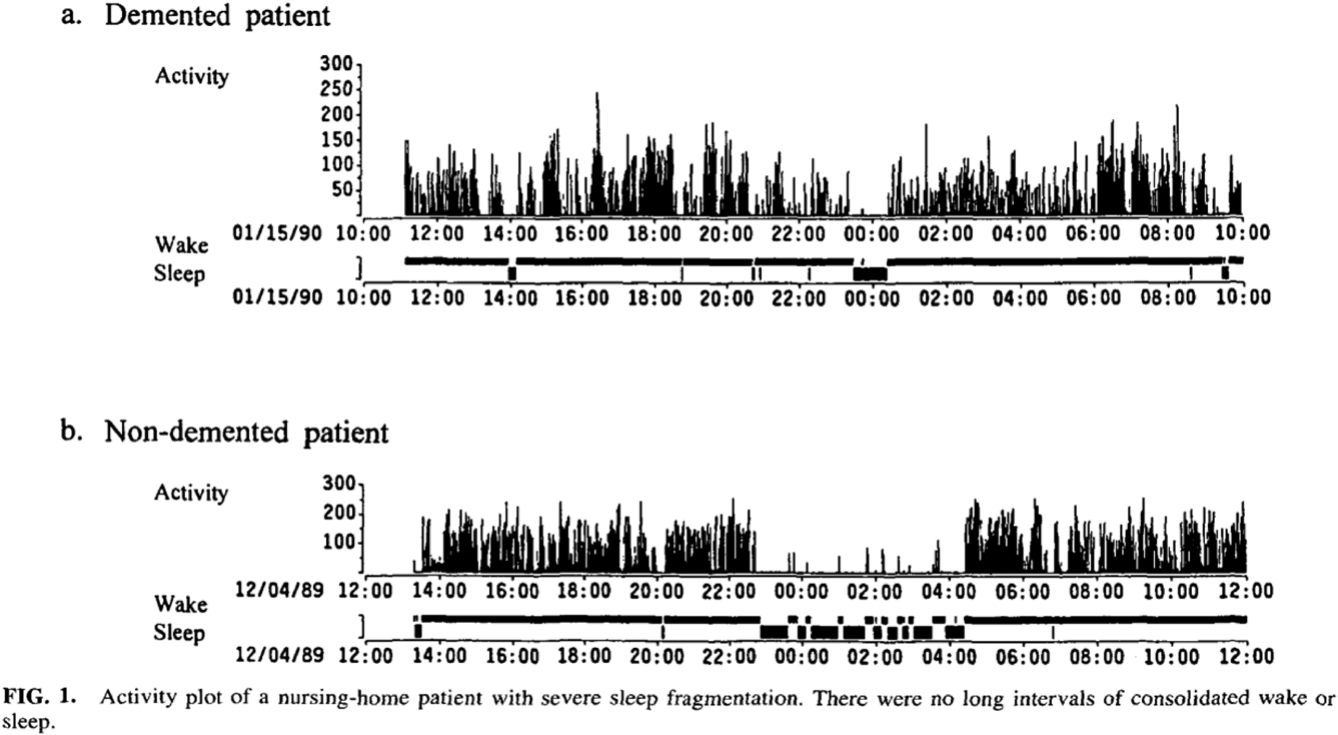
Activity plot of a nursing-home patient with sever sleep fragmentation. From Ancoli-Israel S, Klauber MR, Jones DW, et al. Variations in circadian rhythms of activity, sleep, and light exposure related to dementia in nursing-home patients. Sleep. 1997;20(1):18-23 [12].

When the TNH ended, I wrote a grant to continue my sleep research in nursing homes and was awarded another NIA grant, entitled, “Sleep Consolidation in a Nursing Home Population.” This research ended up exposing the sleep issues confronted by patients residing in nursing homes. We first recorded sleep (using the Medilog recorder as described above) in 200 residents. Results showed that in recordings averaging 15.5 hours, the patients were asleep for just under 8 hours and awake for about 7.5 hours. To obtain that amount of sleep, the patients spent an extended time in bed during the day, averaging no more than 39.5 minutes of sleep per hour in any hour of the night, and 50% woke up at least 2 to 3 times per hour (see Figure 3). So although nursing home patients slept on average only one hour longer than independently living elderly, they had to spend substantially more time in bed to obtain the same amount of sleep [10–12]. And in a 24-hour period, they were never asleep and never awake for a full hour [12, 13].

## Light and Sleep Consolidation

For me, the next obvious step was to examine whether we could “fix” the sleep fragmentation seen in this population. In the next set of studies, we switched from the Medilog recorder to the Actillume (Ambulatory Monitoring, Inc.) which was one of the earliest actigraph devices to record wrist activity and light [14]. We studied 77 patients in the nursing home and found that 47% of the severely demented patients and 20% of the mild-moderate dementia or no dementia group were never exposed to bright light (defined as above 1000 lux). The median number of minutes of bright light for the severely demented group was 1 minute while for the mild-moderate or no dementia group, it was 9 minutes. The combination of all our data confirmed that sleep was extremely fragmented with very little bright light exposure. Our data also showed that patients exposed to higher average illumination levels during the day had more consolidated sleep at night, even when controlling for the level of dementia [15]. So we designed a light treatment study to attempt to consolidate sleep in this group of patients. It was already known that bright light could improve sleep and circadian rhythms, but no one had tried bright light treatment in nursing home patients. We were the first.

We randomized 77 nursing home residents, with a mean Mini-Mental State Examination of 12.8 +/- 8.8 (range 0-30)—suggesting that the majority had some dementia—to one of four treatments: Evening Bright Light, Morning Bright Light, Daytime Sleep Restriction, or Evening Dim Red Light. Light was administered via a Brite-Lite box (Apollo Light System, Orem, UT) placed 1 meter from a patient’s head within a 45-degree visual field, often on top of the television. Patients could read, eat, converse, or watch television during light treatment sessions. As in the above study, sleep and circadian activity rhythms were recorded with the Actillume. Each treatment protocol lasted 10 days plus a baseline period.

Those in the Evening Bright Light group were exposed to 2500 lux from 5:30 pm to 7:30pm, immediately before their standard bedtime. Patients in Morning Bright Light group were exposed to 2500 lux from 9:30 a.m. to 11:30 a.m. Patients in the Evening Dim Red Light were exposed to less than 50 lux red light from 5:30 p.m. to 7:30 p.m. A staff member sat with the patients during each 2-hour light treatment period to ensure that the patients remained awake and did not wander away from the light box. I always joked that we should have recorded my staff’s sleep and circadian activity rhythms as well.

For patients in the Daytime Sleep Restriction group, one staff member accompanied each patient for 6 hours during the day, 9:00 a.m. to noon and after lunch from 2:00 p.m. to 5:00 p.m. The task of the staff members was to ensure that patients did not doze off or fall asleep during this time, thereby restricting sleep during the day.

**Figure 4.**
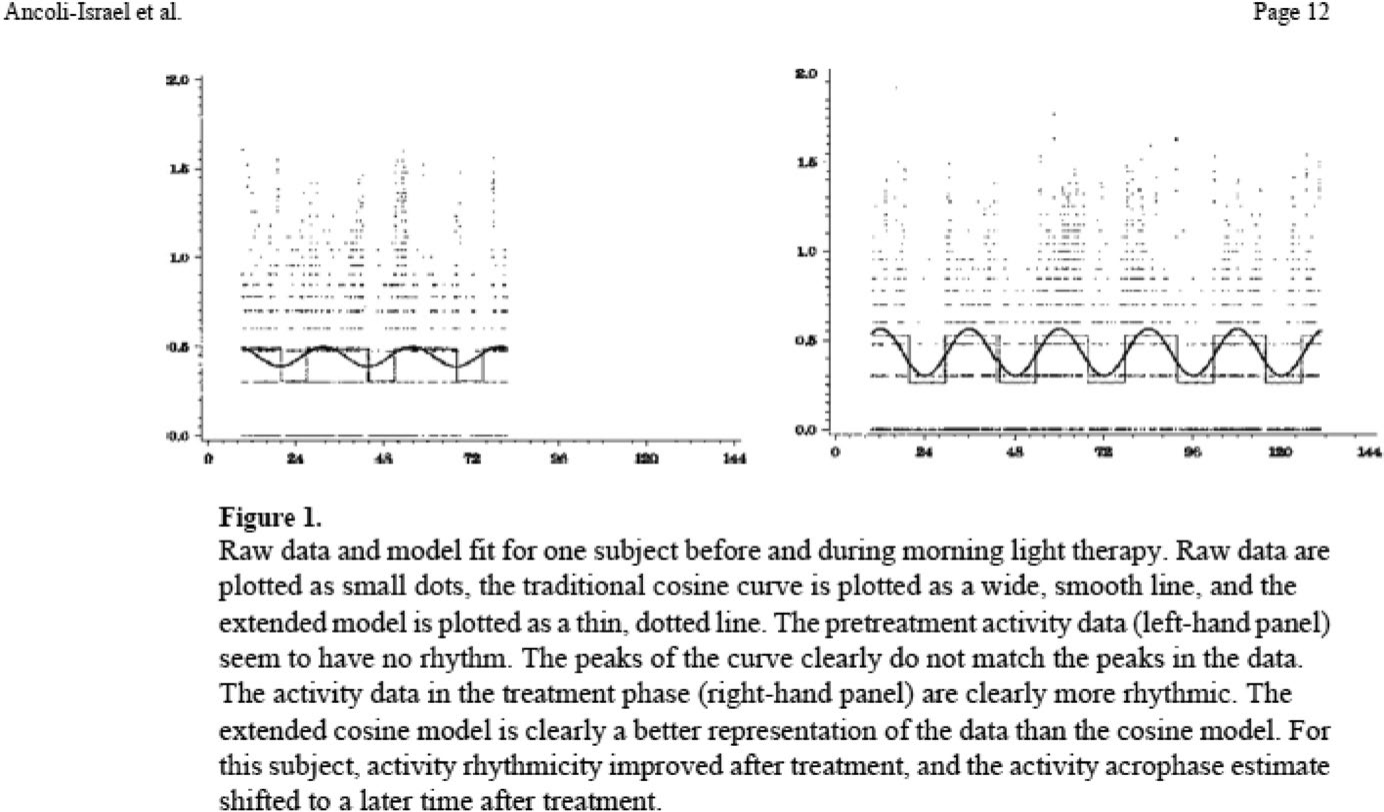
Raw actigraphy data from a nursing home patient during light therapy. From Ancoli-Israel S, Martin JL, Kripke DF, Marler M, Klauber MR. Effect of light treatment on sleep and circadian rhythms in demented nursing home patients. *J Am Geriatr Soc*. 2002;50(2):282-289. doi:10.1046/j.1532-5415.2002.50060 [16].

The results showed no improvements in nighttime sleep or daytime alertness in any of the treatment groups. However, the Morning Bright Light group delayed the peak of the activity rhythm (acrophase) and increased the mean activity level (mesor). In addition, subjects in the Morning Bright Light group had improved activity rhythmicity during the 10 days of treatment (see Figure 4). Therefore, we concluded that while increased light exposure, whether in the morning or evening, did not improve measures of nighttime sleep or daytime alertness, results suggested that morning bright light might delay circadian rhythms and improve circadian rhythm quality in nursing home residents [16, 17]. An additional finding was that the morning bright light advanced the acrophase of the agitation rhythm by over 1.5 hours and was associated with improved caregivers’ ratings [18].

## Sleep Apnea in the Nursing Home

**Figure 5:**
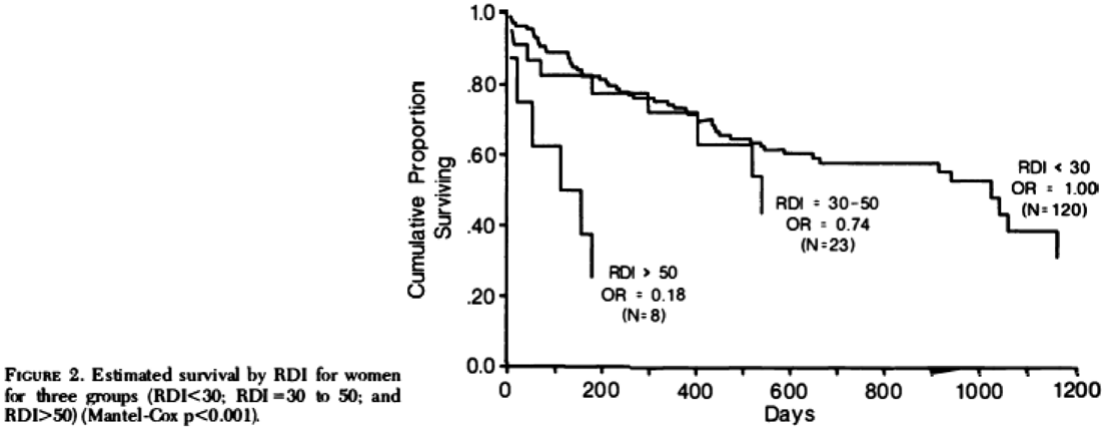
Estimated survival by RDI; from reference Ancoli-Israel S, Klauber MR, Kripke DF, Parker L, Cobarrubias M. Sleep Apnea in Female Patients in a Nursing Home. *Chest*. 1989;96(5):1054-1058. doi:10.1378/chest.96.5.1054^19^

Since we were also recording respiration in some of our studies, we were able to examine the prevalence of sleep apnea in this nursing home population. We had data on 233 patients. The prevalence of sleep apnea (defined as an apnea-hypopnea index ≥5 or respiratory disturbance index [RDI] ≥5) was 70%. We then followed these patients to examine the association of sleep apnea with mortality. The results showed a gender effect with women with sleep apnea having a worse survival rate than men (see Figure 5). In addition, in both genders, those with sleep apnea had a greater tendency to die in their sleep. These results showed that sleep apnea was an extremely significant risk factor for mortality in elderly women who were in poor health [19].

As happens in research, these studies led to more ideas. We realized that most of our patients in the nursing homes had Alzheimer’s disease. We wondered, what might be the association between dementia and sleep apnea? We administered objective neurocognitive tests and found that 96% of the patients had dementia. When compared to the 70% with sleep apnea, we found that the sleep apnea was significantly correlated with all sub-scales on the dementia rating scale. There were significant differences between those with mild-moderate apnea or no apnea and those with severe apnea. In particular, items reflecting attention, initiation and perseveration, conceptualization, and memory tasks on the dementia rating scale distinguished between those with and without severe sleep apnea. Among those patients with no depression, all patients with severe sleep apnea were also severely demented. Our data suggested that there was a strong relationship between dementia and sleep apnea when the sleep apnea and dementia were severe. Although causality could not be inferred from associations, our hypothesis was that sleep apnea caused deficits in brain function, possibly due to global effects rather than any particular cortical or subcortical structure [20].

## Treating Sleep Apnea in Patients with Dementia

For me, the next obvious study that needed to be done was to examine whether treating sleep apnea in these patients would have a positive effect on cognition. To do that, we had to move out of the nursing home into the community. This was the first report of a treatment study in patients with Alzheimer’s disease and sleep apnea. We conducted a randomized, double-blind placebo-controlled clinical trial of 40 patients with mild-moderate Alzheimer’s disease and sleep apnea (defined as an AHI≥10 based on a full night of home polysomnography). Patients were randomized to either 6 weeks of CPAP treatment (which we called the tCPAP group) or 3 weeks of placebo CPAP (which we affectionally called CRAP) followed by 3 weeks of real CPAP (called the pCPAP group). Complete neuropsychological testing was conducted at baseline, after 3-weeks and after 6-weeks.

There were no significant differences in cognition between the first 3-weeks of treatment in the tCPAP group and the three weeks of placebo in the pCPAP group. However, when data from the 3 weeks of treatment in each group were combined, affording a larger sample size, there was modest but significant improvement in cognition from pre- to post-CPAP treatment [21]. This study also resulted in other important findings, including showing that treating these patients with CPAP for three weeks resulted in reduced AHI, improved SaO_2_, deeper sleep (including reduced N1 sleep, arousal index, and WASO, and increased N2 and REM sleep), and reduced daytime sleepiness [22, 23]. It was important to note that these patients with Alzheimer’s disease were able to tolerate the CPAP, using it for an average of 4.8 hours a night, about the same as clinic patients with sleep apnea. Those who were most adherent to CPAP had fewer depressive symptoms, suggesting that treating depression may improve CPAP compliance [24].

But perhaps one of the most important findings in my mind resulted from our ability to compare data from five patients from the treatment study who continued using CPAP after the end of the treatment study (CPAP+) with 5 patients who chose to discontinue CPAP use (CPAP-). Even with the small sample size, sustained CPAP use resulted in moderate-to-large effect sizes in the stabilization of depressive symptoms and daytime sleepiness, and improved sleep quality. But most importantly, sustained use of CPAP slowed the progression of Alzheimer’s disease [25].

## Cancer, Sleep, Fatigue, and Circadian Rhythms—a few of our findings

Since I was one of the first to use actigraphy, and we used the Actillume made by Ambulatory Monitoring, Inc. (AMI), I became good friends with Bill Gruen, founder and president of AMI. Bill would often fly from New York to visit me in San Diego. He always came with his briefcase overflowing with reprints of papers he thought were most important as his love was the scientific challenge. One day, he handed me some papers written by Dr. Francis Levi, on circadian rhythms in cancer. Among other findings, Dr. Levi and his colleagues reported that if the timing of chemotherapy was administered based on the patient’s circadian rhythms, one could give higher doses with fewer side effects. I was intrigued by these findings, but, I thought, we know so little about circadian rhythms in cancer. And thus my other line of research began.

My first cancer grant, funded in the year 2000 by the National Cancer Institute (NCI), was designed to examine fatigue, sleep, and circadian rhythms in breast cancer. The original study was designed to look at women newly diagnosed with breast cancer and study them before they began radiation therapy, and again during therapy. But the oncologist that was collaborating with me left UCSD and so I found other oncologists, first Dr. Vicky Jones and then Dr. Barbara Parker, who specialized in chemotherapy. So, as often happens in research, the design was changed to women about to start chemotherapy.

Previous studies had shown that women with breast cancer undergoing chemotherapy experienced both disturbed sleep and fatigue, but these studies had all been done either just during chemotherapy or in survivors. My belief was that to understand what was happening during chemotherapy, one had to know what these parameters looked like before chemotherapy. We were therefore one of the first (if not the first) to study these women for three days *before* the start of their cancer treatment, for the first three days of weeks 1, 2, and 3 of cycle 1 and of cycle 4 of chemotherapy. One of our first research publications in the area of cancer described these symptoms, fatigue, sleep disturbance, and circadian rhythms, during that baseline, pre-chemotherapy period. We used questionnaires for the fatigue and subjective reports of sleep, and actigraphy for objective measures of sleep and of circadian activity rhythms (CARs). The results suggested that these women already experienced poor sleep and fatigue before the beginning of chemotherapy. Although their CARs were robust during this baseline period, those women with more delayed rhythms experienced more daily dysfunction secondary to fatigue. We concluded that these data suggested that strategies to improve disturbed sleep and to phase-advance circadian rhythms prior to initiation of chemotherapy may be beneficial in improving daily function in breast cancer patients [26]. I later suggested that a combined treatment of bright light and cognitive behavioral therapy for insomnia, both started before the start of chemotherapy might be the best treatment approach. This was an idea I always wanted to pursue, an idea that I was not able to get funded before I retired (more on this later), an idea that I would throw out at the end of every talk I gave, hoping that someone somewhere would take up that mantle. Finally, a group in Australia, led by Joshua Wiley, did just that. But I jump ahead of myself.

**Figure 6.**
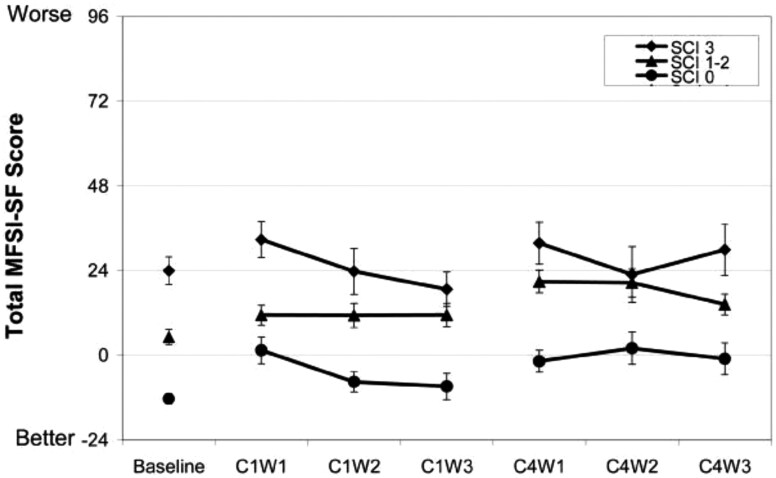
Total Multi-dimensional fatigue scale—short form (MFSI-SF) Score by Cycle/Week. All groups experienced more fatigue during treatment compared to pre-treatment (baseline). During treatment, the group with more symptoms pre-chemotherapy continued to report worse fatigue than the other two groups [27].

Another important finding from this study was the idea of looking at the symptom cluster of sleep disturbances, fatigue, and depression, rather than looking at each individually. The women were divided into three groups based on the number of symptoms they experienced before the start of chemotherapy (i.e. no symptoms, 1–2 symptoms, or all three symptoms) and a symptom cluster index (SCI) was computed. All the women reported worse sleep, more fatigue, and more depressive symptoms during treatment compared with baseline, however, those women with a higher SCI (i.e. more symptoms pre-treatment) continued to experience worse symptoms during treatment compared with those who began with fewer symptoms (see Figure 6). We again concluded that treatment strategies for this symptom cluster should begin before the start of chemotherapy [27].

**Figure 7.**
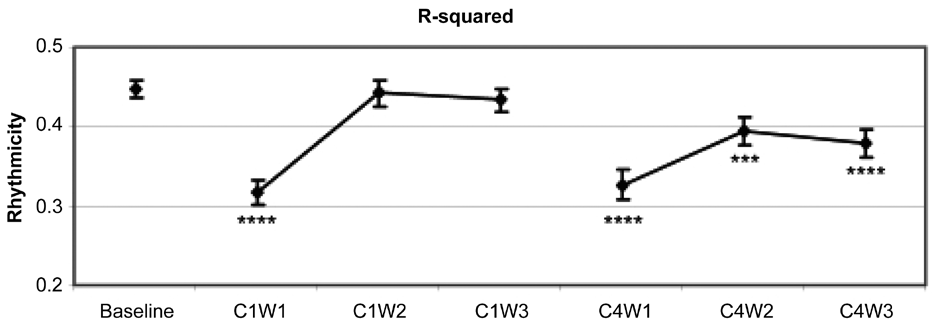
Mean and standard deviations for R-squared (strength of the circadian rhythm) showing that that the first administration of chemotherapy was associated with transient disruption of sleep-wake rhythm, while repeated administration of chemotherapy resulted in progressively worse and more enduring impairments in sleep-wake activity rhythms [28].

I was lucky enough during that time to have Dr. Josée Savard, an expert in sleep, fatigue, and cancer in her own right, do a sabbatical in my lab. Josée, while she was working on her own manual for behavioral therapy for insomnia for cancer patients, examined some of our CAR data. Previous studies had shown that sleep-wake activity rhythms in cancer patients show little distinction between night and day suggesting circadian disruption. However we were able to assess the longitudinal course of sleep-wake activity rhythms before and during chemotherapy. The results showed that compared to baseline, with the exception of acrophase, all circadian activity rhythm variables examined were significantly impaired during the first week of both chemotherapy cycles (i.e. cycle 1 and cycle 4). Although the circadian variables approached baseline values during weeks 2 and 3 of cycle 1, most remained significantly more impaired during week 2 and week 3 of cycle 4, suggesting that the first administration of chemotherapy was associated with transient disruption of sleep-wake rhythm, while repeated administration of chemotherapy resulted in progressively worse and more enduring impairments in sleep-wake activity rhythms (see Figure 7) [28].

**Figure 8.**
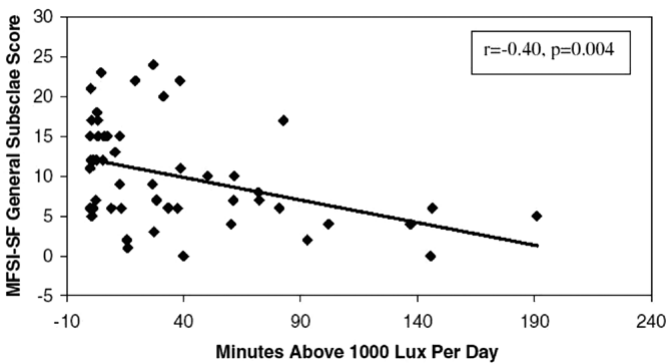
Scatterplot of general fatigue (MFSI-SF) and duration of bright light exposure (minutes > 1000 lux/day) during cycle 1 week 1. More general fatigue was significantly correlated with fewer minutes of > 1,000 lux light exposure [29]

Another finding of this study, which was the impetus of some of my subsequent research in cancer, was that increased fatigue during chemotherapy in these women with breast cancer was significantly associated with decreased bright light exposure within both cycle 1 and cycle 4 (see Figure 8). There were also significant correlations between changes in light exposure and changes in fatigue within the first 2 weeks of each cycle. My co-authors and I concluded that “Although the cause and effect of exacerbated fatigue and decreased light exposure cannot be confirmed by the current study…These results suggest the need for prospective intervention studies of light therapy for breast-cancer-related fatigue” [29]. And that is exactly what we did next.

We received funding from the California Breast Cancer Foundation and a Litebook Society for Light Treatment and Biological Rhythms (SLTBR) research award to examine the effect of light treatment during chemotherapy on fatigue, sleep, mood, and quality of life in women with breast cancer. Women with newly diagnosed breast cancer were randomized to either bright white light (BWL) or dim red light (DRL; < 50 lux). They were instructed to use their light box for 30 minutes every morning upon awakening throughout four cycles of chemotherapy. Light boxes were donated by Litebook, Inc. and modified to include an integrated meter that recorded the time and duration the light box was on, allowing for the collection of compliance data. Data on fatigue, sleep, mood, and quality of life were collected before the start of chemotherapy and light treatments, during cycle 1 and 4 chemotherapy week, and during the last week of cycles 1 and 4. The results showed that the DRL group showed increased fatigue during chemotherapy, as expected, but the BWL treatment prevented fatigue from getting worse during chemotherapy compared to baseline [30]. Our hypothesis that overall fatigue would improve with bright light treatment was not supported, but I was happy with our findings that light would at least prevent fatigue from getting worse. When we looked at CAR and at mood and quality of life, we found that the morning bright light did in fact prevent deterioration of these variables [31, 32]. These results suggested that morning bright light could be a useful intervention during chemotherapy for fatigue in breast cancer. When I retired in 2012, several colleagues continued this line of research, including Drs. William Redd, Heiddis Valdimarsdottir, Lisa Wu and Ali Amidi, and Sheila Garland and her colleagues, using the same light protocol, and confirming many of our results. And they graciously included me as a co-investigator in those studies.

But that wasn’t the end of my interest in sleep and cancer. The next line of research was to examine longitudinal changes in my variables of interest, i.e. sleep, fatigue, and CAR. The next cancer grant, also funded by NCI, looked at how women did before chemotherapy, during chemotherapy, and one year after the end of chemotherapy. This time we had a comparison group of age-, ethnicity- and education matched women with no cancer. Our results showed that compared to the non-cancer women, women with breast cancer spent significantly more time napping, had worse sleep and less sleep at night, more depressive symptoms, more fatigue, more disrupted activity rhythms, and worse quality of life at baseline. By the end of cycle 4 of chemotherapy, compared both to the controls and to their own values at baseline, the patients had significantly more depressive symptoms, worse sleep quality, more fatigue, worse quality of life, more disrupted activity rhythms, and longer naptimes. At one year post-chemotherapy, the patients returned to their baseline levels but were still worse than controls on all measures [33].

The third line of research in cancer patients grew out of the increased interest in what at the time was called “chemobrain.” In our longitudinal prospective one-year follow-up study, we also collected neuropsychological data, both subjective and objective. Interestingly, we found that while subjectively the women reported worse cognition both during and one year after chemotherapy, objective testing showed no change in cognitive function from baseline to the end of cycle 4, but did show improvement at one year. However, the controls showed a learning effect while the patients did not, suggesting they may have been experiencing some learning deficit, perhaps due to the acute effects of chemotherapy [34].

In the same study, we were able to look at predictors of cognitive decline. The best predictor both at the end of chemotherapy cycle 4 and at one year was CAR robustness [34]. Future research still needs to examine whether bright light therapy, which would improve the CAR, would also have a positive effect on cognitive fog associated with chemotherapy.

## Other Research

There is not enough room in one paper to review everything I did. I also studied sleep in Parkinson’s disease, schizophrenia, sleep and race, sleep (and health) in caregivers of Alzheimer’s patients, and more.

But my most fun paper was totally different. In the mid-1990s, I was privileged to be part of a two-year course on leadership in the Jewish community. As part of that course, we studied the bible, the New Testament, the Koran, and much more. While reading the bible (Old Testament), I began to notice many references to sleep. And that led to the most fun I had writing a paper on what the Hebrew tradition has to say about sleep and sleep disorders. Turns out that many of the things we think we discovered, were already referred to thousands of years ago, and that “there is nothing new under the sun.” I encourage you to read that paper [35].

## Collaborate, Collaborate, Collaborate—Colleagues

I was so fortunate to have many wonderful colleagues over the years. I was always a firm believer in working with others, sharing ideas, and thus letting research ideas flourish. I worked closely at UCSD with (in alphabetical order): Drs. Wayne Bardwell, Jody Corey-Bloom, Joel Dimsdale, Chris Gillin, Igor Grant, Dilip Jeste, Mel Klauber, Dan Kripke, Leah Levi, Jose Loredo, Paul Mills, Atul Malhotra (and all his colleagues from his lab), Loki Natarajan, Barbara Parker, Barton Palmer, Tom Patterson, Georgia Sadler, Michael Ziegler, just to name some. Within my own laboratory I had so many wonderful staff working with me over my 45-year career that I can’t mention them all (and I hope they forgive me). But two that stand out for the outstanding work they did with me, and for me, are William Mason and Dr. Lianqi Liu.

I also got to collaborate with many colleagues outside of UCSD and those collaborations are some of the reasons I have so many publications. One recommendation I have for junior investigators is—collaborate, collaborate, collaborate. Many of my publications stem from working on the MrOs and SOF studies under Dr. Katie Stone and all the other investigators involved in those studies (including Kristine Yaffe and Yu Leng and too many others to name and too many publications to list). Suffice it to say that I was a co-author on close to 130 publications from that collaboration.

And then there were the collaborations with the American Academy of Sleep Medicine where I got to be part of the original working group for the newly developed scoring manual for sleep [36].

I also had the honor to both work and publish with others in our field (again in alphabetical order and just to name a few): Drs. Ruth Benca, Mary Carskadon, Don Bliwise, Dan Buysse, Peter Hauri, Meir Kryger, Marge Moline, Charles Morin, Barbara Phillips, Tom Roth, Josee Savard, Michael Vitiello, and Phyllis Zee, all who became my good friends as well as colleagues.

I do have to mention one more colleague and friend, Dr. Andrew Monjan. Andy was the Chief of the Neurobiology of Aging Branch of the Division of Neuroscience within the National Institute on Aging at NIH, and he was a huge advocate and supporter of mine. Sometimes I think it is really thanks to him that I was continually funded by NIA from 1980 until my retirement in 2012. Although I suppose I had something to do with it.

It is especially rewarding when your colleagues become your friends. With all the business traveling we do, we often see many of our colleagues more frequently than our friends at home. And it is those friendships that help make what we do so much fun.

## Women in Sleep

**Figure 9.**
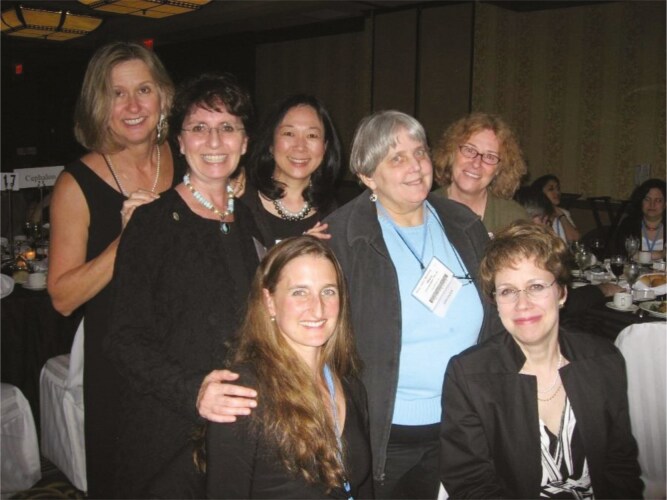
Some of my women colleagues: Standing: Barbara Phillips, Sonia, Phyllis Zee, Mary Carskadon, Terry Young; seated: Jennifer Martin, Ruth Benca.

**Figure 10.**
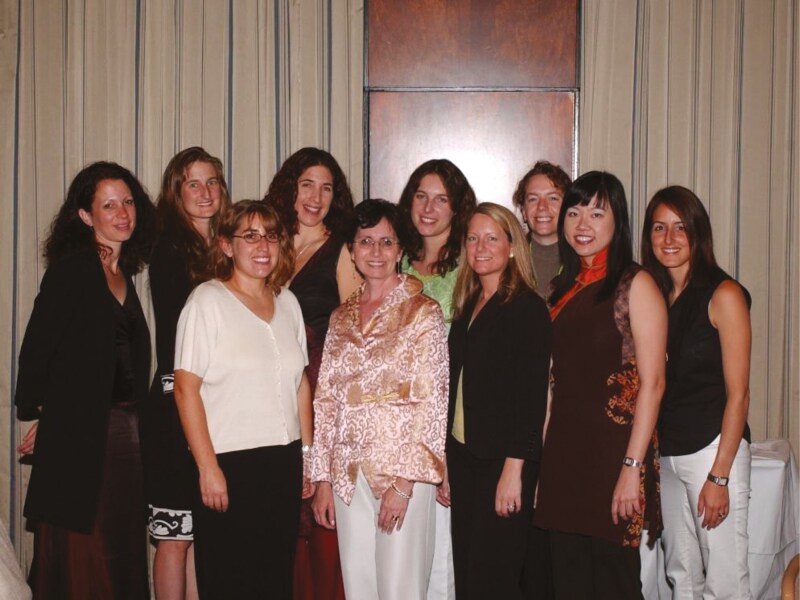
Me with some of my women mentees in 2005 (Tamar Shochat, Jennifer Martin, Jana Cooke, Liat Ayalon, Sonia, Lavinia Fiorentino, Judy Profant, Tricia Haynes, Mei Sian Chong, Mairav Cohen-Zion.

I attended my first sleep meeting in 1978. At that time there were very few women in sleep. There was Ros Cartwright. There was Mary Carskadon. There was Pat Prinz, Irene Tobler, Anna Wirz-Justice. There was me. And there were a few others. But not many. And so we banded together, over the years, adding more women into our sphere. We looked out for each other. We worked hard to make sure women were represented on the boards and in the programs. And we stuck together. Women are much better represented these days, although we still have to work hard for equal representation. One of my fondest memories is the dinners we would have the sleep meetings, just women, supporting each other and being friends (see Figure 9 and 10).

## My Greatest Legacy

**Figure 11.**
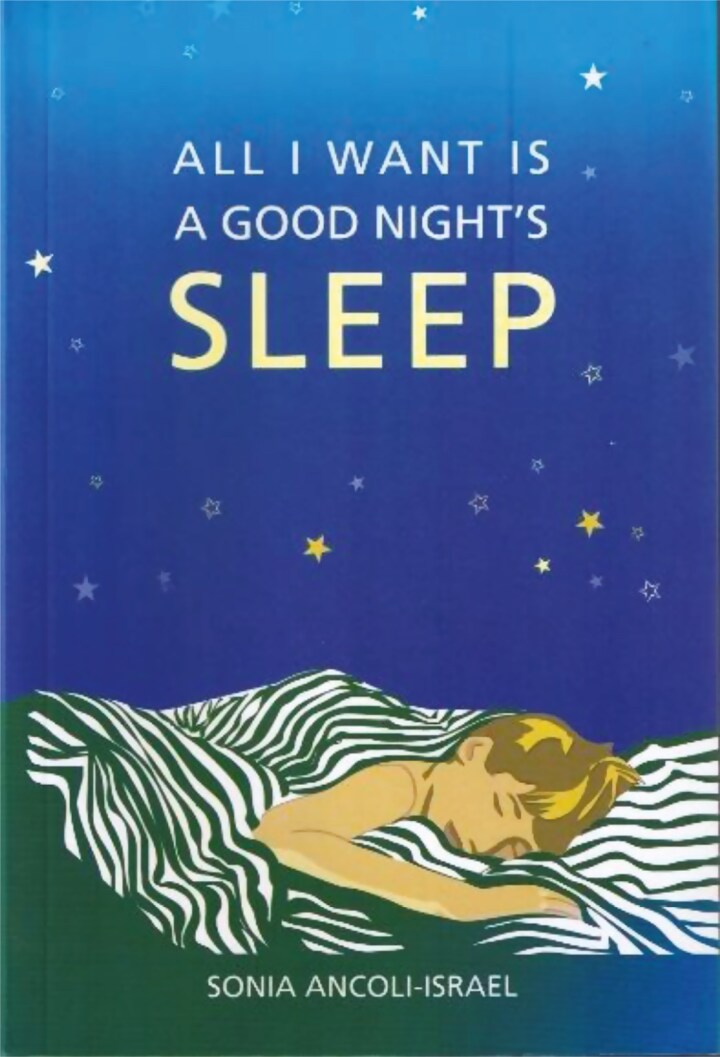
My book for the lay public.

I am extremely proud of the work I did over the 45 years of my career. My overarching goal was always to improve quality of life by improving sleep, particularly in older adults, in patients with neurogenerative disease (Alzheimer’s and Parkinson's), schizophrenia, insomnia, and cancer, whether through teaching, through my research or through treating patients. I wrote a book for the lay public to help them learn how to sleep better (see Figure 11). All that is part of my legacy.

But my greatest legacy is my students. We are all continuously learning, acquiring wisdom, and part of that continuous learning is continuous teaching, because the best way to learn is from your students. I taught high school students and undergraduate students. I taught graduate and post-graduate students. I taught PhDs and MDs, psychologists and psychiatrists and neurologists, pulmonary physicians, and even surgeons. It just goes to show what an interdisciplinary field sleep is and how we get to make an impact in so many ways.

**Figure 12.**
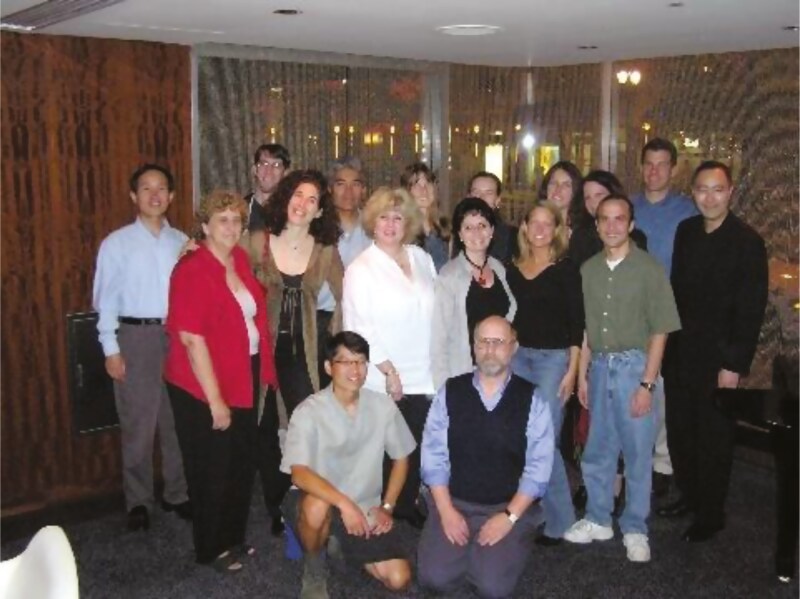
APSS dinner 2004.

**Figure 13.**
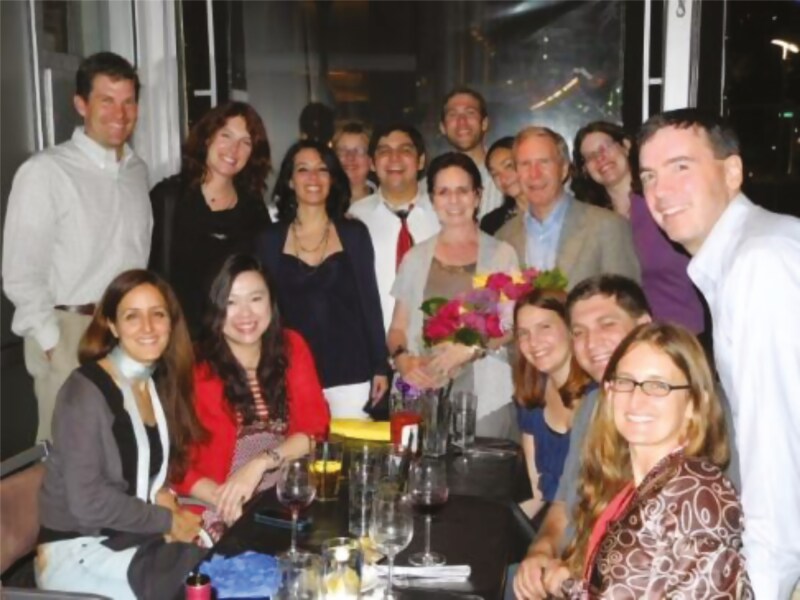
APSS dinner 2012—the year I retired.

I was so fortunate to have such wonderful graduate students, clinical practicum students, and post-doctoral students. Most of them stayed within our field of sleep, and many have become leaders—and mentors—in their own right. Again, in alphabetical order: Liat Ayalon, Mei Sian Chong, Mairav Cohen-Zion, Jana Cooke, Naima Covassin, Sean Drummond, Lavinia Fiorentino, Rena Fox, Phil Gehrman, Michael Grandner, Tricia Haynes, Clete Kushida, Jose Loredo, Jennifer Martin, Polly Moore, Rachel Morehouse, Ariel Neikrug, Sara Nowakowski, Henry Orff, Ruth Pat-Horenczyk, Judy Profant, Michelle Rissling, Tamar Shochat, Carl Stepnowsky and so many others I have mentored officially and unofficially—too many to name. And each year, at APSS, I would hold a dinner for every former or current student (see Figures 12 and 13). I think some of my former students now do that for their students and so the tradition lives on.

I always tried to convey to my students the passion and conviction I felt about our wonderful field. My students kept me on my toes, they forced me to keep up, they helped me do my research; write papers, and chapters and grants. My research group represented a “family” in which, I always hoped, a sense of belonging was evident to all of them. And I always told them - once a mentor, always a mentor with no expiration date. I always thanked them for the respect and trust they placed in me and for all THEY taught ME.

**Figure 14.**
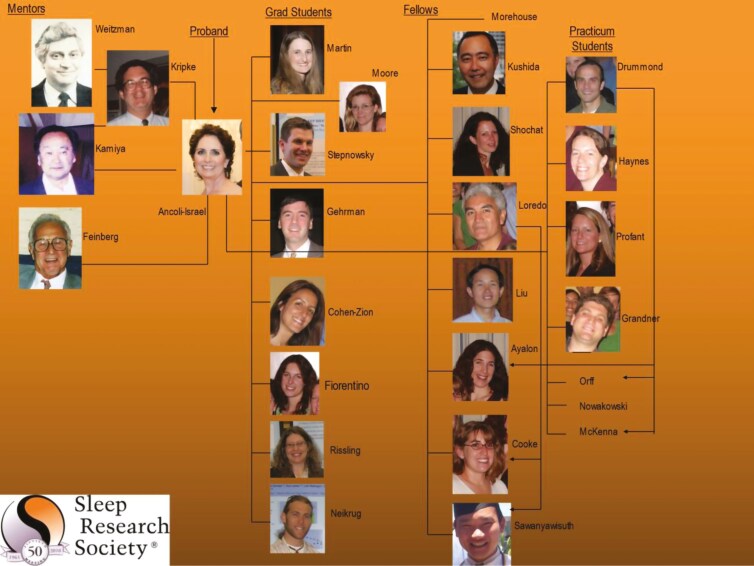
My professional family tree.

For the SRS’s 50^th^ anniversary, I was asked to chair the celebration. As part of that, I made a “sleep” family tree (see Figure 14). We are all so connected.

**Figure 15.**
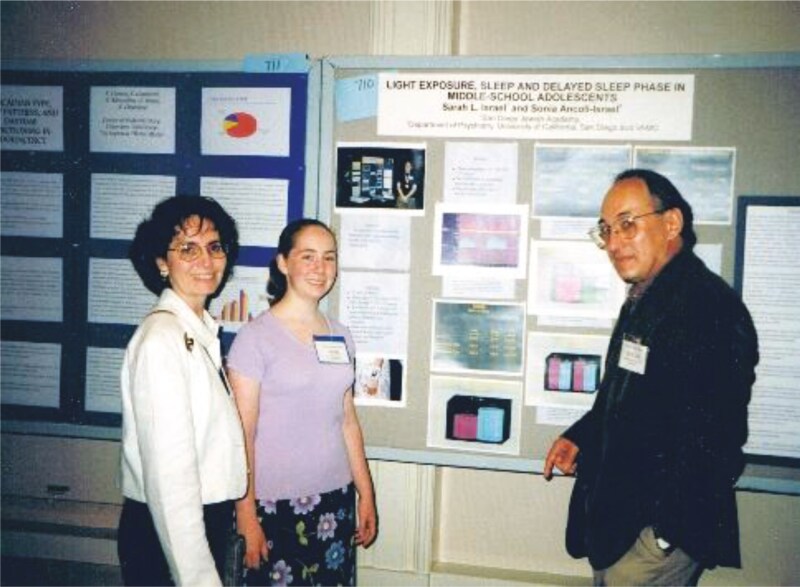
Sonia, Sarah Israel Gimbel and Andy Monjan.

**Figure 16.**
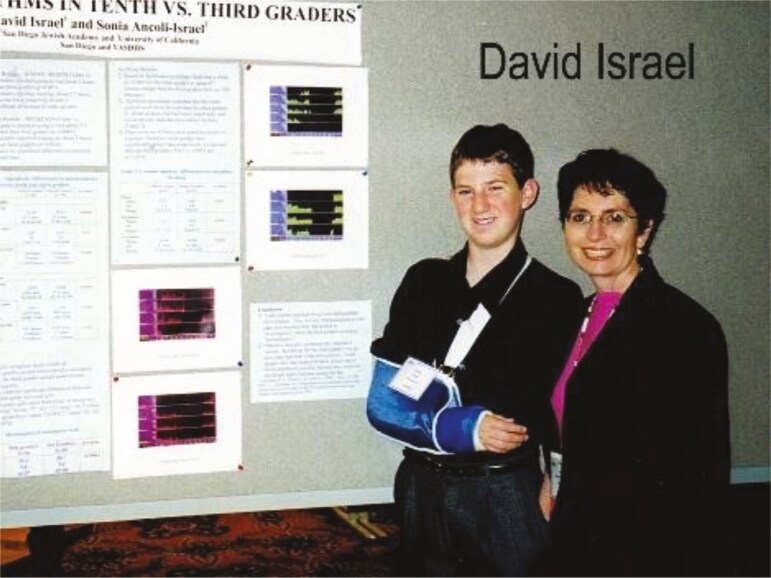
David Israel and Sonia.

I have to add something about two more “students” I’m so very proud of. Both my children, when they were in middle school and had to do a science fair project, chose to do them in sleep, using actigraphs they borrowed from my lab. I think they were each the youngest to have poster presentations at both Association of Professional Sleep Societies (APSS) annual meeting (see Figure 15) and the Society for Light Treatment and Biological Rhythms (SLTBR) annual meeting (see Figure 16).

## Balancing and Juggling

There are different aspects to life and, as I have always tried to teach my students, to be really successful you have to learn to balance—to successfully juggle—all aspects of your life. In our field, we talk about yin/yang, sleep/wake. I want to add work/play. To be successful you need to have a life outside of work, and for me, that life was my family. I nurtured my students beyond their academic sleep training, teaching them how to do just that. On the one hand, I encouraged them to work hard and to always aspire to move forward in their academic careers, and on the other hand, I would remind them that family life comes first, and that their responsibilities as a spouse, parent, or child are more important than their careers.

The only way I could do this balancing act was by setting my priorities, with my family coming first. To this day, my children haven’t let me forget that I turned down a chance to be on Oprah so I could be at my daughter’s sixth-grade math field day competition (which by the way, she won). They still have not forgiven me all these years later, but for me, it was a no-brainer—I have no regrets.

## Fun Anecdotes

With a career spanning 45 years, there had to be some good stories to tell. I will tell you a few of them.

In 2003, the American Academy of Sleep Medicine began administering an exam in Behavioral Sleep Medicine (BSM). I told the group in charge that after close to 30 years in the field, and being one of the founders, I was *not* going to take another exam. They politely told me that no one was being grandfathered (grandmothered?) in and that if I wanted to teach BSM to my students, I had to take the exam and be certified. Plus, they wanted me to set an example for others. So, I grudgingly took the exam, telling them I would only do it if they gave me certificate number 1! I sat in the large room with my colleagues who were also taking the exam. Ed Stepanski was our proctor. Suddenly, I started to laugh. Ed came over to me to ask what was so funny. I pointed to a question and asked him, “Do you want me to give you the correct answer, or the answer I know you want?” He looked at the question, looked at me, and said, “Give me the answer we want.” The question was something like, “The prevalence of sleep apnea defined as AHI>5 in the older adult is …25%.” I knew this came right from my work. But I also knew that the prevalence of 25% referred to AI > 5; the prevalence of AHI > 5 was closer to 60%. So I gave them the answer they wanted, although it was wrong. I’m pretty sure they dropped that question. And by the way, my certificate was number 5, not number 1!

**Figure 17.**
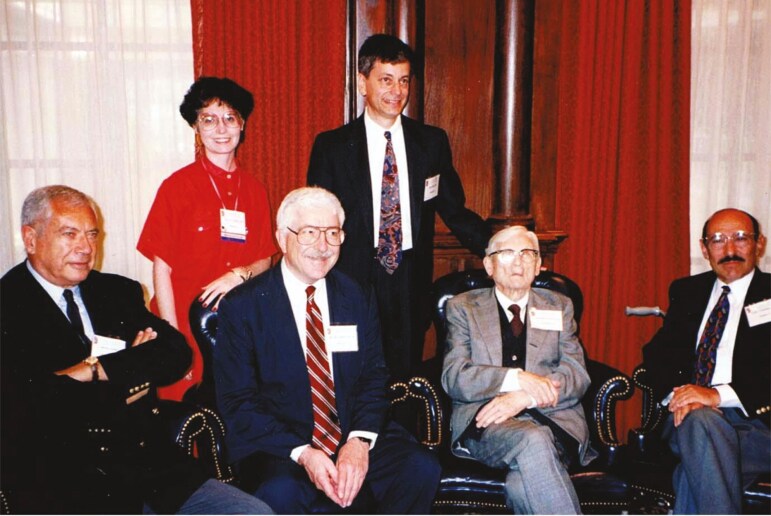
Michel Jouvet, Bill Dement, Nathanel Kleitman, Eugene Aserinsky (all seated) with Jerry Siegel and me.

In 1995, the sleep meeting was in Nashville, TN and it was a celebration of Nathaniel Kleitman’s 100th birthday. I was on the program committee and Jerry Siegel, who was the program chair, and I were lucky enough to be included in a private reception that took place right after the opening ceremonies. The four giants of sleep were all there, and I cherish this photograph with them—all of whom are no longer with us: Michel Jouvet, Bill Dement, Nathaniel Kleitman, and Eugene Aserinsky (see Figure 17).

**Figure 18.**
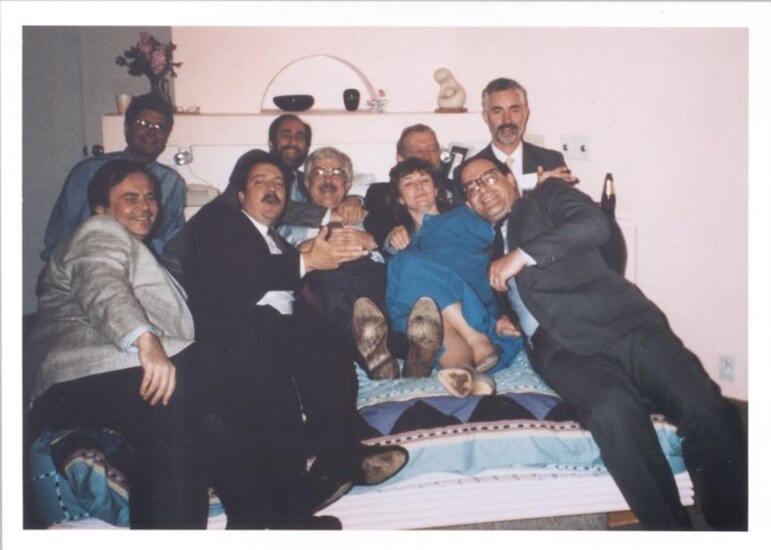
The APSS board meeting in my bedroom. From left to right: Chip Reynolds, Mike Chase, Phil Westbrook, Jim Walsh, Bob McCarley, Merrill Mitler, Bill Dement, Sharon Keenan, Tom Roth.

Another anecdote is from 1988 when the APSS meeting was in San Diego. My San Diego colleagues, including Chris Gillin, Dan Kripke Merrill Mitler and I, threw a party in my backyard. But it was also the night of the AASM board meeting. No one wanted to miss our party, so they held the board meeting in my bedroom (see Figure 18). This is one of my favorite pictures.

**Figure 19.**
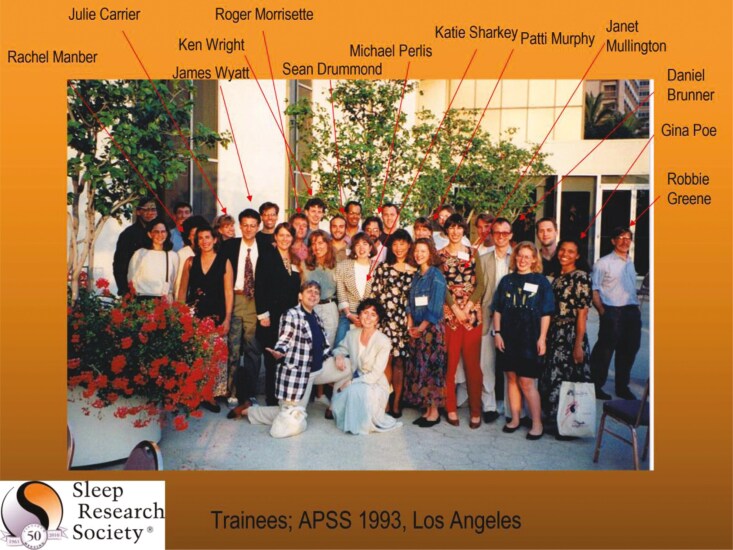
Mary Carskadon and me with the SRS trainees.

And speaking of favorite photos, this is another one I love. I showed it at the SRS’s 50^th^ anniversary which I chaired. Mary Carskadon was the first to oversee the SRS trainee program, now called the Training and Education Advisory Committee (TEAC) and I was second, serving as the SRS Program Chair for Trainees from 1992 - 1996 and on TEAC from 1997 through 2000. Here are Mary and me with some of our early trainees, all of whom went on to become mentors and successful researchers and clinicians (see Figure 19).

## Giving Back

It is one thing to do your research, collaborate, and see patients. But it is also important to give back to the community that supports you. I sat on NIH study sections. I was on many editorial boards and reviewed countless journal papers for countless journals. I was president of SLTBR from 2000-2002. I was president of the SRS from 2004-2005. I was the Secretary of the founding board of the National Sleep Foundation. I was on the program committee for APSS and on the APSS executive board. In 1985 I helped write the initial clinical polysomnography exam (the pre-cursor for the current AASM certification exam). And so much more. For example, for many years, along with Martica (Tica) Hall and Gina Poe, I undertook recording the SRS “Conversations with Our Founders,” a series of video interviews with many of the earliest people in our field, many of whom are no longer with us. I encourage you to watch those videos, available on the SRS website. And now, I am just finishing my stint as Associate Editor of the Living Legends section of Sleep Advances. How honored am I to actually be one of the people asked to write this story.

Within UCSD I was the chair of the Behavioral Medicine Tract, UCSD/SDSU Joint Doctoral Program in Clinical Psychology from the inception of the program until my retirement in 2012 and I sat on many other UCSD committees.

Give back!

## Awards

**Figure 20.**
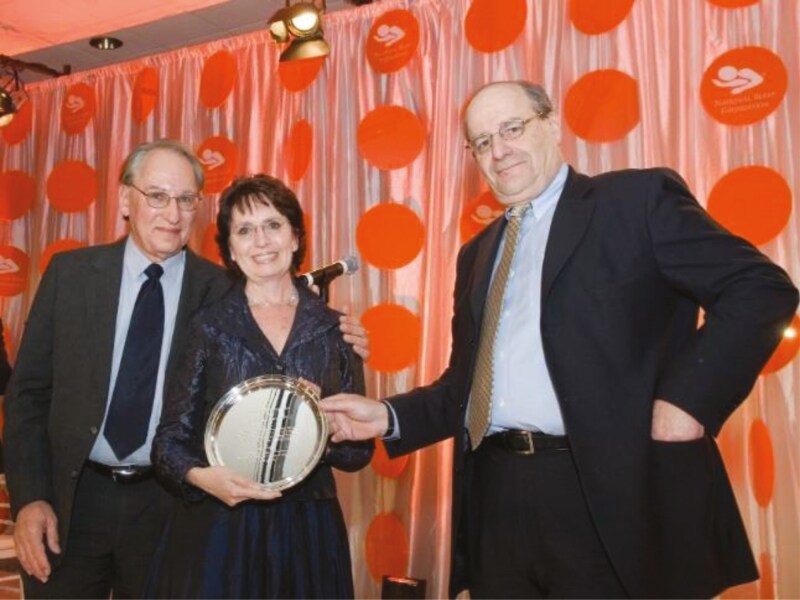
Andy Monjan and Tom Roth presenting me with the National Sleep Foundation Life Time Achievement Award.

As I mentioned, I am so proud of my science and of my students. But being recognized for one’s work is also a source of pride. My first award was the National Sleep Foundation (NSF) Lifetime Achievement Award in 2007. I had nominated many people in past years, so when I got an email from the NSF with the subject line of the award, I assumed it was about that. I literally was in shock when I read that I had been selected. I had no idea—none—that I was at a place in my career for such an accomplishment, or that there were people who recognized the significance of my work. The NSF gala that year was in a large banquet hall in a hotel in Washington DC and people came from all over the world to honor me. They even gave me a standing ovation. I continued to be in shock. I had chosen two people to present the award to me—Dr. Tom Roth and Dr. Andy Monjan (see Figure 20). I already wrote about Andy Monjan and how important he was in my life and in my career. And Tom was the one who first invited me to join the NSF board back when the NSF was just starting, and who pulled me into so many other professional endeavors.

**Figure 21.**
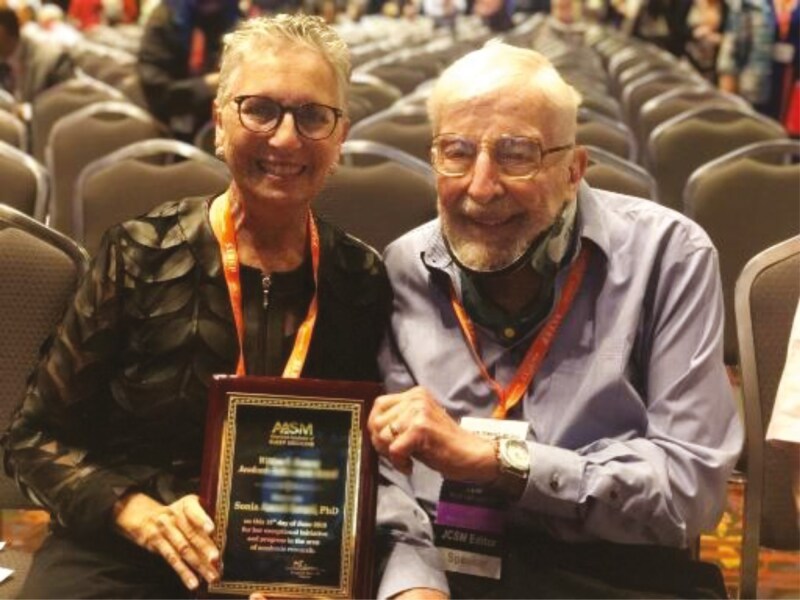
Bill Dement and me when I received the American Academy of Sleep Medicine William C. Dement Academic Achievement Award.

Over the next years, I won almost every award our field has to give. In 2007 I was the third recipient (after Mary Carskadon and Bill Dement) to win the Sleep Research Society Mary A. Carskadon Outstanding Educator Award. This award for teaching excellence was conceived during my term as President of the SRS because, as I have already mentioned, teaching has always been of utmost importance to me and it was fitting—and a no brainer—to name it after Mary. You can’t imagine my surprise when I learned that I was that year’s recipient. I then went on to be the one of the first recipients of the Society of Behavioral Sleep Medicine’s Career Distinguished Achievement Award, later renamed after Peter Hauri. I was always sad that my plaque didn’t have his name—my colleague and long-time friend. Over the next few years, I received the Sleep Research Society Distinguished Scientist Award and the American Academy of Sleep Medicine William C. Dement Academic Achievement Award. I was the last one to get the award in Bill Dement’s name with him still in the audience (see Figure 21). As part of my thank you speech when accepting the award that year, I said, “I have been retired for 7 years, but I never felt like I was left behind on the sidelines. The fact that you recognize my work makes me realize that I helped build a foundation in this field, and I am so gratified watching all of you who continue to build on my work. I treasure that kind of recognition.” And I still cherish that recognition.

There were three more years, after my retirement, where I received recognition much to my great surprise. In 2022 I was named one of the Best 1000 Female Scientists in the World ranking #1 at UCSD, 107 in the US, and 161 in the world. These rankings were based on the number of citations of our work. In 2023, I was again on that list, still ranking #1 at UCSD but dropping to 111 in the US and 180 in the world. And again in 2024, still ranking #1 at UCSD, but dropping again to 132 in the US, and 203 in the world. Given that I had already been retired for 12 years at that point, I was still very proud that my work was still being cited at such high levels.

Speaking of being retired, I also was awarded the 2024 Dickson Emeriti Professorship award which honors UCSD emeritus professors whose outstanding research, scholarly work, teaching, or public service activities have continued since retirement. I clearly don’t understand the definition of retirement.

## Travel

I always loved to travel. As academicians, we get to go to international meetings and get invited to speak all over the world. Andy and I always added vacation time to the work time. And since Phyllis Zee and her husband, Ben, were at all those same meetings, the four of us would travel together. When we had a meeting in South Korea, we added a trip to Vietnam. When we had a meeting in Bangkok, we added Cambodia. When we had a meeting in Rome, we added Israel. And the list goes on and on.

I am also an amateur but avid photographer, so I started a travel blog with my stories and photographs (www.JourneysWithSonia.com). Check it out. These days I write about travel instead of writing grants. Ah, retirement.

## Retirement

I retired in 2012 when my husband Andy was diagnosed with Posterior Cortical Atrophy, a rare form of early-onset Alzheimer’s disease. He was 60 years old. I wanted to make the most of the time we had left together, and so I stepped back from work. We had a few more good years, and then I became the caregiver. The irony of the fact that I studied sleep in Alzheimer’s all those years, and had studied the effect of being a caregiver was not lost on me. I was practicing what I had preached.

Right before I retired, I got back the “pink sheets” from my last grant submission (the grant reviews used to come on pink paper, thus the name). It was an idea I always wanted to pursue, a study using a combination of light therapy and CBT-I in women with breast cancer, that would begin even before the start of their chemotherapy. I was convinced that this would prevent their symptoms of fatigue, poor sleep, and disrupted circadian rhythms from getting worse during their treatment. I got great reviews but since I was a seasoned researcher, the score just missed being funded. I’m still waiting to see if I was right.

My retirement is not a complete retirement. I continue to be emerita—collaborating with colleagues, giving talks, and consulting when asked. I have been on the executive board of the UCSD Center for Circadian Biology and am involved with what has become one of the most eminent circadian conferences in the world. I get to do all the fun stuff and say no to those things I don’t want to do anymore. I have the best of all worlds.

## Family

I can’t write about my career without talking about my family. I already mentioned that my husband Andy had Alzheimer’s Disease. He died in October of 2020, just short of his 69th birthday. Andy was the most supportive husband I could have asked for, always encouraging me and putting up with my late nights and my extensive travel. He was my heart and my soul. He not only encouraged me to believe in my dreams, he also believed in them.

Andy and I had two children—Sarah who became a neuroscientist and David who became an attorney. Sarah is married to Jeremy, a Rabbi/Cantor, and they have two children, Ari and Maya. David is married to Taylor, also an attorney, and they also have two children, Orli and Bekin. They are the light of my life. I am so lucky that they all live in San Diego, and I get to see them all the time. My children were (and are) all very supportive of my work, my travels, and my life. They put up with me working all hours of the day and night, but I think I was their role model in how to be a successful professional parent. Now they are each wonderful working parents in their own right.

## Conclusion

I had the privilege of starting my career at a time when sleep was a burgeoning field, and little was known about sleep in aging or its implications for health. For me, the world revolves around sleep. Sleep touches everything, which is why it is so crucial to keep educating the world about the importance of a good night’s sleep. By writing this paper and looking back over my work, I realize now that I helped build a foundation in this field, and I am so gratified watching my colleagues and my former students continue to build on my work and to keep educating the world.

## Notes

*This paper is part of the Living Legends in Sleep Research series, which is sponsored by Idorsia Pharmaceuticals and Jazz Pharmaceuticals.*
